# BMI variability and incident diabetes mellitus, Tehran Lipid and Glucose Study (TLGS)

**DOI:** 10.1038/s41598-022-22817-6

**Published:** 2022-11-01

**Authors:** Ladan Mehran, Pouria Mousapour, Davood Khalili, Leila Cheraghi, Mohammadjavad Honarvar, Atieh Amouzegar, Fereidoun Azizi

**Affiliations:** 1 Endocrine Research Center, Research Institute for Endocrine Sciences, Shahid Beheshti University of Medical Sciences, Tehran, Islamic Republic of Iran; 2 Prevention of Metabolic Disorders Research Center, Research Institute for Endocrine Sciences, Shahid Beheshti University of Medical Sciences, Tehran, Islamic Republic of Iran; 3 Department of Biostatistics and Epidemiology, Research Institute for Endocrine Sciences, Shahid Beheshti University of Medical Sciences, Tehran, Islamic Republic of Iran

**Keywords:** Endocrinology, Endocrine system and metabolic diseases, Type 2 diabetes

## Abstract

Previous epidemiologic studies debated the association of body mass index (BMI) trends with cardiovascular disease and mortality. This study aimed to evaluate the association of BMI variability and slope with the incidence of Type 2 diabetes mellitus (T2DM) in a sex-stratified 15.8-year follow-up in the population-based Tehran Lipid and Glucose Study (TLGS). Of 10,911 individuals aged 20–60 years, 4981 subjects were included and followed for 15.8-years. The slope coefficient of BMI in the linear regression model represented individuals’ BMI trends up to the incidence of DM. The root mean squared error (RMSE) of the BMI linear trend was selected to reflect BMI variability through six follow-ups. Cox proportional hazards regression was used to investigate the association of the baseline BMI, BMI slope and RMSE with the incidence of T2DM among men and women. Multivariable-adjusted HRs of T2DM for each SD increment in BMI slope was 1.18 (95% CI: 0.94–1.48, p = 0.161) in normal weight men and 1.26 (95% CI: 1.10–1.44, p = 0.001) in overweight and obese men. However, in women, each SD increment in BMI slope increased the risk of T2DM with a HR of 1.19 (95% CI: 1.01–1.40, p = 0.039) in normal weight, and 1.14 (95% CI: 1.08–1.19, p < 0.001) in women with BMI ≥ 25 kg/m^2^. In men with a baseline BMI ≥ 25 kg/m^2^, BMI-RMSE was associated with a decreased risk of T2DM (HR: 0.71, 95% CI: 0.53–0.93, p = 0.015). Baseline BMI was not associated with the risk of diabetes in men and women. Positive BMI slope is associated with the development of diabetes in both sexes. The association of BMI variability with incident T2DM differs according to sex and baseline BMI. BMI variability is associated with a lower risk of T2DM in overweight and obese men. BMI variability in women and baseline BMI in both gender are not related to the risk of T2DM.

## Introduction

Type 2 diabetes mellitus (T2DM) is an independent risk factor for a spectrum of non-communicable diseases and a leading but preventable cause of reduced quality of life and life expectancy. According to the Global Burden of Disease (GBD) report, the worldwide prevalence of T2DM dramatically increased from approximately 333 million persons in 2005 to approximately 435 million persons in 2015^1^, with the T2DM associated mortality rate increasing from 1.2 million to 1.5 million annually^2^.

Obesity is strongly associated with prevalent T2DM and T2DM associated mortality^3^ and is a major established but modifiable predictor of T2DM in the general population^4^. Weight reduction has been considered essential to prevent T2DM and its disabling and life-threatening complications^5,6^. Nevertheless, T2DM is not limited to obese individuals, and overweight or normal weight individuals may also experience the disease, suggesting that T2DM patients comprise heterogeneous groups concerning their body mass index (BMI) status during the years before diagnosis.

Unlike a single BMI measurement, BMI slope, defined as the overall BMI trend, and BMI variability, defined as intra-individual variability of BMI overtime-points, might be able to provide a dynamic longitudinal evaluation of body weight trajectories. Previous epidemiologic studies have indicated strong associations between increased weight variability and hypertension, cardiovascular disease, and mortality^7^. However, results from studies evaluating the association of weight variability with T2DM have not been consistent. In the most recent review on 253,766 participants, weight variability increased the risk of developing T2DM; however, the association did not exist in men, individuals aged ≤ 60 y., and normal weight and obese individuals^8^.

Understanding the heterogeneity in the weight change patterns before the incidence of T2DM can provide important implications for implementing prevention strategies. The purpose of the present study was to evaluate the association of baseline BMI, BMI slope, and variability with the incidence of T2DM in a sex-stratified 15.8-year follow-up in the Tehran Lipid and Glucose Study (TLGS).

## Materials and methods

### Study design

TLGS is a prospective population-based cohort launched in 1999 to determine the risk factors for non-communicable diseases among a representative Tehran urban population. Participants of TLGS were recruited from 1999 to 2001 by multistage cluster random sampling methods. The Data collection continued for 20 years up to 2018, at approximately three years intervals. The study design, sampling methodology, and data collection have been previously published^9^.

### Study population

For the current study, a total of 10,911 individuals aged 20–60 years were enrolled in the study baseline. We excluded individuals with T2DM at the baseline (n = 853), missing T2DM status (n = 669), glomerular filtration rate (GFR) < 30 ml/min/1.73m^2^ (n = 8), those with incident T2DM within the first two follow-up phases (n = 361), pregnancy (n = 249), regular use of systemic corticosteroids (n = 346) and incident cancer during the study follow-up (n = 106). We then excluded individuals who missed follow-up (n = 2324). From the remaining 5995 individuals, 1014 individuals were also excluded due to missing anthropometric information. Finally, the total valid sample of 4981 subjects was included in the analysis.

### Clinical and biochemical measurements

Data of the TLGS, including interviews, anthropometric and biochemical measurements, were recorded and collected by trained general practitioners. Information regarding age, sex, medical history, smoking habits, medication use, education level and family history of T2DM was obtained through pretested interview questionnaires. Data on physical activity was collected using the Persian-translated forms of the Lipid Research Clinic (LRC) questionnaire in the first^10^ and the Modifiable Activity Questionnaire (MAQ) in subsequent follow-ups^9^; therefore, the data was rescored on the metabolic equivalent of the task scale (METs), with METs < 600 min-week^-1^ considered as low physical activity^11^.

Blood pressure (BP) was measured twice in a seated position after 15 min resting using a standard mercury sphygmomanometer. For laboratory tests, venous blood samples were drawn from all participants between 07:00 and 09:00 a.m., after 12 to 14 h of fasting. High-density lipoprotein (HDL) in plasma was measured after precipitation of the apoB-containing lipoproteins with phosphotungstic acid. Plasma triglyceride (TG) and total cholesterol (TC) concentrations were assessed using the enzymatic colorimetric method (Pars Azmoon, Inc, Iran). Low-density lipoprotein (LDL) was calculated using the Friedwald formula. Fasting plasma glucose (FPG) and two-hour post-challenge plasma glucose (2-hPG) were assessed using glucose oxidase by the enzymatic colorimetric method. Serum creatinine values were quantified by the standard colorimetric Jaffe_Kinetic reaction method (Pars Azmon Inc., Iran). Details on anthropometric and laboratory measurements and techniques are available in previously published papers^9^.

### Definition of terms

Smoking status was defined as 1. Smokers (daily or occasional use of any tobacco product) 2. Non-smokers (ex-smokers or those who have never smoked). Education status was classified as 1. Illiterate/primary school (less than six years); 2. Below diploma /diploma (6–12 years), and 3. Higher than diploma (more than 12 years).

T2DM was defined as FPG ≥ 126 mg/dl, 2 hPG ≥ 200 mg/dl, or participants using any antihyperglycemic drugs; and impaired glucose tolerance (IGT) was described as 2-hPG (75 g oral glucose tolerance test) between 140 and 199 mg/dl^12^. Dyslipidemia was defined as TG ≥ 200 mg/dl, TC ≥ 240 mg/dl, HDL < 40 mg/dl, or consumption of lipid-lowering drugs^13^. Hypertension was defined as systolic blood pressure (SBP) ≥ 140 mmHg, diastolic blood pressure (DBP) ≥ 90 mmHg, or taking antihypertensive drugs^14^. BMI was calculated as weight (kg) divided by height (m^2^). Subjects with BMI < 25, 25–29.9 and ≥ 30 kg/m^2^ were defined as normal, overweight, and obese, respectively.

### Baseline BMI, BMI variability and slope

Our predictor variables for this analysis were the baseline BMI values, slope, and variability (BMI-RMSE). Instead of weight, BMI-based residuals were used for the analyses since the scale of residuals varies with height. The slope coefficient of BMI in the linear regression model was used to represent an individual's BMI trend during the time up to the incidence of T2DM. The root mean squared error (RMSE) of the regression line (predicted BMI trend) was selected as the measure to reflect the variability of the BMI within each individual, based on at least three BMI measurements. The term “BMI variability” is to indicate variations in BMI values considering the absolute changes of BMI, without considering the direction of changes; however, to consider the final direction of weight change, BMI slope was adjusted as a confounding factor. To reflect BMI variability, BMI-RMSE was calculated using the following formula:

$${RMSE}_{i}=\sqrt{\frac{{\sum }_{j=1}^{H}{\left({BMI}_{i,j}-{\widehat{BMI}}_{i,j}\right)}^{2}}{N} ,}$$

$${\mathrm{BMI}}_{\mathrm{i},\mathrm{j}}=\mathrm{Actual} \; \left(\mathrm{observed}\right) \; \mathrm{longitudinal \; BMI},$$

$${\widehat{\mathrm{BMI}}}_{\mathrm{i},\mathrm{j}}=\mathrm{Estimated }\;\left(\mathrm{fitted}\right)\;\mathrm{longitudinal \; BMI},$$

$$\mathrm{H}=\mathrm{Number \; of \; measured \; BMIs},$$

$$\mathrm{i}=1,..,\mathrm{N},$$

$$\mathrm{j}=1,\dots ,6.$$

We also investigated the association of BMI variability with T2DM incidence using other indices, including variability independent of the mean (VIM), standard deviation (SD), and coefficient of variation (CV).

### Statistical analysis

Baseline characteristics were presented as mean ± SD for normally distributed variables, median (interquartile) for skewed variables, or frequency for categorical variables. The Kolmogorov–Smirnov test was used to check the normality of data distribution. *T* test and Mann–Whitney *U* test for continuous variables and the Chi-Square test for categorical variables were performed to compare baseline characteristics between men and women.

**Figure 1:**
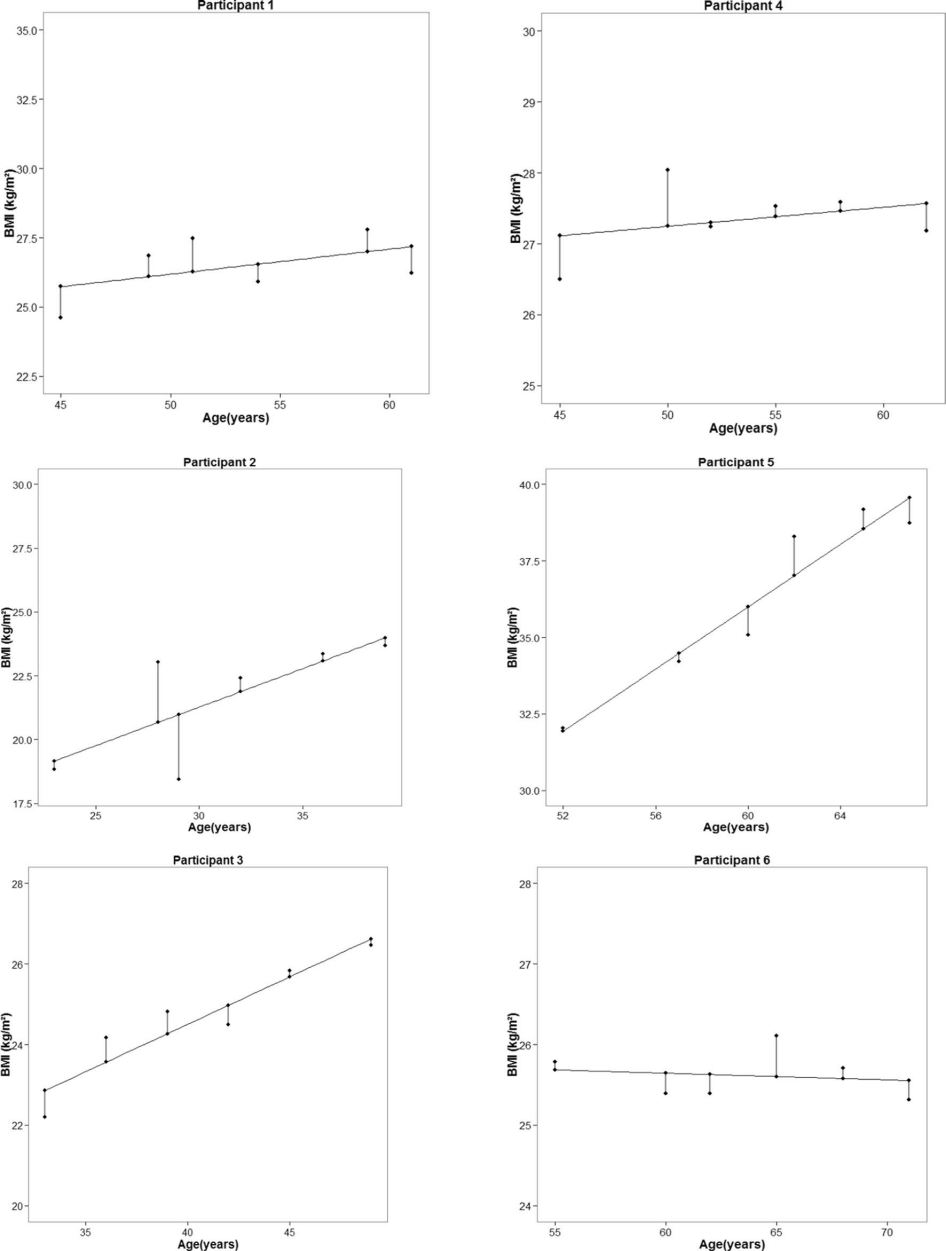
Observed and fitted values of BMI change and residuals from 6 randomly selected male and female participants (Left: Males, Right: Females).

We considered BMI at the baseline, the linear slope of BMI during the follow-ups, and BMI variability (RMSE) over the BMI trend line as predictor variables for the current study. The BMI slope and variability during the time up to the incidence of T2DM were calculated using a simple linear regression model. A separate model was estimated for each participant, in which BMI and follow-up examination time were considered dependent and independent variables, respectively. The slope coefficient of the model represented the linear trend of a participant's BMI change in direction and magnitude. The RMSE for each person (the residuals of BMI variation in time points) was calculated to represent the magnitude of BMI variations. The linear trend of BMI changes and residuals in each time point are illustrated for six randomly selected male and female participants in Fig. 1.

The crude incidence rate (95% CI) of T2DM was calculated by dividing the number of new cases of T2DM by person-years at risk for each sex and the whole population. Survival time was considered from entry into the study to the first incidence of T2DM. The censoring time for a participant was the time from the entry into the study to the following time points, whichever happened first: loss to follow-up, death from any cause, or the end of the study without having T2DM.

Unadjusted and adjusted Cox proportional hazards regression models were used to investigate the association of baseline BMI, BMI-RMSE and BMI slope with the incidence of T2DM. We have checked for potential effect modification by sex and baseline BMI. As there was a significant interaction effect between baseline BMI (cut-off of 25 kg/m^2)^ and BMI-RMSE (p = 0.032) and between sex and BMI-RMSE (p = 0.017), the analyses were performed separately in men and women with BMI < 25 kg/m^2^ and BMI ≥ 25 kg/m^2^. Two models were performed in the current study; Model 1 was adjusted for age, baseline BMI, BMI slope, and RMSE. Model 2 was adjusted for age, baseline BMI, BMI slope, RMSE, family history of diabetes, education level and smoking status. Proportional hazard assumptions in Cox models were checked using Schoenfeld’s residuals test^15^; all the proportionality assumptions were generally appropriate. The statistical analysis was conducted using SPSS software version 24.0 (SPSS Inc, Chicago, IL, USA) and STATA 14 (StataCorp, college station, TX, USA). All *p*-values were reported as two-sided, and a p-value < 0.05 was considered significant.

### Ethical approval

This study was performed according to the ethical principles of the Helsinki Declaration, and all procedures involving human subjects were approved by the National Research Council of the Islamic Republic of Iran (IR.SBMU.ENDOCRINE.REC.1398.103), the Human Research Review Committee of the Endocrine Research Center, Shahid Beheshti University, Tehran, Iran. Written informed consent was obtained from all subjects.

## Results

A total of 4981 (43% male) adults without diabetes at the baseline were analyzed. A comparison of the baseline characteristics between men and women of the study population is illustrated in Table 1. Mean values of the BMI measures (26.9 vs. 25.5 kg/m^2^) and the prevalence of obesity (24.8% vs. 12.9%) were significantly higher in women than in men. No difference was observed between men and women in the mean age and family history of T2DM.

**Table 1:** Baseline characteristics of the study population.

	Males (n = 2138)	Females (n = 2843)	p value
Age, years	37.53 ± 10.54	37.33 ± 10.65	0.498
BMI, kg/m^2^	25.57 ± 4.00	26.98 ± 4.76	< 0.001
**BMI subgroups, n (%)**			
< 25 kg/m^2^	956 (45.2)	991 (35.5)	< 0.001
25–29.9 kg/m^2^	887 (41.9)	1108 (39.7)	
≥ 30 kg/m^2^	272 (12.9)	691 (24.8)	
Hypertension, n (%)	727 (34.0)	895 (31.5)	0.062
IGT, n (%)	1112 (52.0)	1304 (45.9)	< 0.001
FPG, mg/dl	89.51 ± 8.57	87.81 ± 8.58	< 0.001
2-hPG, mg/dl	97.54 ± 26.03	105.61 ± 25.15	< 0.001
Dyslipidemia, n (%)	1590 (74.4)	1492 (52.5)	< 0.001
Family history of diabetes	544 (25.4)	729 (25.6)	0.896
Smokers, n (%)	1067 (49.9)	205 (7.2)	< 0.001
**Education level, n (%)**			
Illiterate or primary	333 (15.6)	795 (28.0)	< 0.001
Secondary	1363 (63.8)	1728 (60.8)	
Higher	440 (20.6)	318 (11.2)	

Categorical variables are represented as frequency (percent). Continuous variables are represented as mean ± SD.

*SBP* systolic blood pressure, *DBP* diastolic blood pressure, *IGT* impaired glucose tolerance, *FPG* fasting plasma glucose, *2-hPG* 2-h post-challenge plasma glucose, *HDL* high-density lipoprotein, *LDL* low-density lipoprotein.

*Triglycerides levels are reported as median (IQR 25–75).

The median follow-up for the current study was 15.8 years (IQ25-75: 14.1–16.8). 242 and 333 new cases of T2DM were identified among men and women over 31,090.71 and 41,319.00 person-years of follow-up, respectively. Incident rates of T2DM were 7.78 (6.86–8.83) in men and 8.06 (7.24–8.97) in women per 1000 person-years. Table 2 shows the minimum, maximum, mean and the percentiles values of baseline BMI, BMI slope and RMSE in men and women. The corresponding information regarding other BMI variability indices, including SD, CV and VIM, is provided in the Supplementary Table S1.

**Table 2:** The mean, SD and percentile values of BMI variation (RMSE), slope and baseline BMI.

					Percentiles				
	Mean	SD	Minimum	Maximum	5th	25th	50th (median)	75th	95th
**Male**									
Baseline BMI	25.57	4.00	15.12	52.12	19.27	22.86	25.46	28.08	31.88
BMI slope	0.11	1.74	− 6.80	9.68	− 2.49	− 1.01	0.03	1.10	3.13
BMI-RMSE	1.13	0.70	0.2	9.92	0.43	0.70	0.97	1.37	2.33
**Female**									
Baseline BMI	26.98	4.76	16.17	48.04	19.36	23.71	26.71	29.97	35.18
BMI slope	0.15	4.97	− 12.93	24.95	− 6.84	− 3.23	− 0.37	2.89	9.11
BMI-RMSE	1.55	0.85	0.17	8.63	0.59	0.99	1.37	1.90	3.12

Sex- and BMI-stratified HRs and 95% CIs of developing T2DM for baseline BMI, BMI slope and RMSE are shown in Table 3. Age-adjusted HRs of T2DM for baseline BMI, BMI slope and RMSE were 1.21 (95% CI: 1.04–1.40, p-value = 0.011), 1.39 (95% CI: 1.15–1.67, p-value < 0.001) and 1.31 (95% CI: 0.99–1.73, p-value = 0.055) in men with BMI < 25 kg/m^2^, and 1.01 (95% CI: 0.88–1.17, p-value = 0.858), 1.09 (95% CI: 0.99–1.20, p-value = 0.07) and 1.13 (95% CI: 0.76–1.69, p-value = 0.54) in women with BMI < 25 kg/m^2^. However, in participants with BMI ≥ 25 kg/m^2^, the age-adjusted HRs of T2DM for baseline BMI, BMI slope and RMSE were 1.10 (95% CI: 1.06–1.15, p-value < 0.001), 1.25 (95% CI: 1.15–1.35, p-value < 0.001) and 0.87 (95% CI: 0.68–1.11, p-value = 0.258) in men, and 1.13 (95% CI: 1.10–1.16, p-value < 0.001), 1.12 (95% CI: 1.09–1.14, p-value < 0.001) and 1.34 (95% CI: 1.21–1.50, p-value < 0.001) in women, respectively.

**Table 3:** Sex- and BMI-stratified hazard ratios of type 2 diabetes mellitus.

	Baseline BMI		BMI slope		RMSE	
	HR (%95 CI)	*p* value	HR (%95 CI)	*p* value	HR (%95 CI)	*p* value
**Males**						
**BMI < 25**						
Unadjusted	1.25 (1.08–1.44)	0.003	1.13 (0.95–1.33)	0.168	1.23 (0.88–1.72)	0.224
Age-adjusted	1.21 (1.04–1.40)	0.011	1.39 (1.15–1.67)	< 0.001	1.31 (0.99–1.73)	0.055
Model 1	1.16 (0.97–1.37)	0.1	1.21 (0.96–1.52)	0.098	1.28 (0.91–1.8)	0.153
Model 2	1.14 (0.96–1.36)	0.129	1.18 (0.94–1.48)	0.161	1.37 (0.95–1.98)	0.095
**BMI ≥ 25**						
Unadjusted	1.09 (1.05–1.14)	< 0.001	1.19 (1.10–1.29)	< 0.001	0.82 (0.64–1.06)	0.126
Age-adjusted	1.10 (1.06–1.15)	< 0.001	1.25 (1.15–1.35)	< 0.001	0.87 (0.68–1.11)	0.258
Model 1	1.03 (0.96–1.10)	0.351	1.26 (1.10–1.43)	0.001	0.70 (0.53–0.93)	0.015
Model 2	1.02 (0.96–1.09)	0.537	1.26 (1.10–1.44)	0.001	0.71 (0.53–0.93)	0.015
**Females**						
**BMI < 25**						
Unadjusted	1.08 (0.94–1.23)	0.275	1.02 (0.93–1.11)	0.714	1.00 (0.66–1.52)	0.989
Age-adjusted	1.01 (0.88–1.17)	0.858	1.09 (0.99–1.20)	0.07	1.13 (0.76–1.69)	0.54
Model 1	0.84 (0.64–1.09)	0.195	1.19 (1.00–1.40)	0.042	0.84 (0.46–1.51)	0.055
Model 2	0.84 (0.64–1.10)	0.196	1.19 (1.01–1.40)	0.039	0.85 (0.48–1.52)	0.586
**BMI ≥ 25**						
Unadjusted	1.14 (1.11–1.17)	< 0.001	1.09 (1.07–1.12)	< 0.001	1.22 (1.10–1.36)	< 0.001
Age-adjusted	1.13 (1.10–1.16)	< 0.001	1.12 (1.09–1.14)	< 0.001	1.34 (1.21–1.50)	< 0.001
Model 1	0.97 (0.91–1.04)	0.423	1.13 (1.08–1.19)	< 0.001	1.03 (0.88–1.20)	0.727
Model 2	0.97 (0.90–1.03)	0.293	1.14 (1.08–1.19)	< 0.001	1.04 (0.89–1.21)	0.643

Model 1 is adjusted for age, baseline BMI, BMI slope, and BMI-RMSE.

Model 2 is adjusted for model 1, as well as family history of diabetes, education level and smoking.

*RMSE* root mean squared error, *HR* hazard ratio, *CI* confidence interval.

After full adjustments, baseline BMI values had no association with T2DM in any sex and BMI groups. BMI slope increased the risk of T2DM in men with BMI ≥ 25 kg/m^2^ (HR: 1.26; 95% CI: 1.10–1.44, p-value = 0.001) and in women (women with BMI ≥ 25 kg/m^2^, HR: 1.14; 95% CI: 1.08–1.19, p-value < 0.001; women with BMI < 25 kg/m^2^, HR: 1.19; 95% CI: 1.01–1.40, p-value = 0.039). BMI-RMSE was only associated with lower risk of T2DM in men with BMI ≥ 25 kg/m^2^ (HR: 0.71; 95% CI: 0.53–0.93, p-value = 0.015). BMI variability was also investigated using SD, CV and VIM, which all replicated the same results regarding the significance of the association with future T2DM in the fully adjusted model (see Supplementary Table S2 online). In men with BMI ≥ 25 kg/m^2^, the corresponding HRs in the fully adjusted model for SD, CV and VIM of BMI were 0.67 (95% CI: 0.50–0.90, p-value = 0.008), 0.90 (95% CI: 0.82–0.98, p-value = 0.016), and 0.92 (95% CI: 0.85–0.98, p-value = 0.015), respectively.

## Discussion

In a large community-based cohort among Middle Eastern adults without diabetes, we investigated the association of baseline, BMI slope, and BMI variability with T2DM during a median 15.8-year follow-up. BMI variability was negatively associated with the development of T2DM in obese and overweight men; however, no association was found in women. There was no association between the baseline BMI values and the incidence of T2DM in both sexes. BMI slope was the significant independent predictor of T2DM in both sexes.

Higher baseline BMI values are correlated with higher levels of CRP and HbA1C; however, it does not moderate the relationship between BMI slope and these biomarkers^16^. Weight gain is a well‑established risk factor for T2DM, and different weight management strategies, even with modest impacts on weight loss, have produced a significant decrease in T2DM incidence in both obese and non-obese patients^5,17^. The current study showed that baseline BMI, regardless of BMI slope, was not associated with the development of T2DM in men and women, as the association was diminished after adjustment for BMI slope and RMSE. However, a positive BMI slope was associated with the increased risk of T2DM in both genders, independent of the baseline BMI and BMI variability. Therefore, the BMI slope can be considered a more potent predictive factor for T2DM than its baseline value. In line with the current report, in a 10-year follow-up of US male participants aged 40–75, the weight loss and weight gain slopes were significantly associated with decreased and increased incidence of T2DM, respectively^18^. An 11-year follow-up of a representative sample of Chinese adults aged 20–50 demonstrated that BMI slope is a significant predictor for incident T2DM, independent of baseline BMI^19^. In the cohort of Japanese workers aged 35–66 years, the slope in self-reported weight change was significantly associated with the development of T2DM in both sexes^20^. In a recent study on adolescents on the effect of BMI on glucose hemostasis, BMI slope had a positive direct and indirect impact (CRP-mediated pathway) on HbA1c overall baseline BMI levels^16,21^.

Weight variability is reported to have an independent effect on cardiovascular risk, possibly due to increased accumulation of abdominal visceral fat^22^ and elevations in circulatory lipids^23^. Indeed, visceral adiposity is a source of inflammatory markers and adipokines and a well-known predictor of insulin resistance and T2DM. Previous studies reported the association of weight variability with increased total body fatness and adverse effects on lipid profile, insulin sensitivity, and glucose levels in young adults, even in those who maintain normal weight^22–26^. However, further studies are needed to find the mechanisms underlying the association between weight variability and incident T2DM. There is little evidence of the association of weight or BMI variability with the development of T2DM, and the results are controversial^27–31^. In the current study, BMI variability in overweight and obese men was associated with the development of T2DM after adjusting for baseline BMI and BMI slope. In contrast to the current study, the EPIC-Germany cohort reported that weight variability was not an independent risk factor for T2DM in the adult population, however in the subgroup analysis in those with net weight gain, weight variability posed a stronger risk of diabetes than those with stable body weight^32^; this study was limited by the retrospectively self-recalled reports and differed with the current study regarding the design and definition of weight variability. In line with the present report, a study in Korea showed a protective effect of BMI variability (using ASV) on T2DM in obese (baseline BMI ≥ 25) in contrast to its predictive value in non-obese Korean subjects (baseline BMI < 25)^33^. Also, a recent study on Japanese urban individuals showed that high BMI and body-fat RMSE decreased the risk of T2DM in men; however, the association was unclear in women^34,35^. The probable explanation of the protective effect of BMI variability for T2DM in overweight and obese male participants may be due to the dominant decreasing trend of BMI during frequent up and down weight changes, which again highlights the importance of BMI slope rather than variability in the prediction of T2DM. Also, the negative association between BMI variability and the risk of T2DM might be imposed by lifestyle modification in men with BMI ≥ 25 kg/m^2^. Previous literature elucidated the association of body weight variability with impaired glucose metabolism through increasing the adipocyte’s size and the amounts of lipogenic enzymes, palmitic, myristic, palmitoleic and stearic acids^36^; conversely, the weight variability might improve glucose metabolism by reducing the amounts of linoleic and α-linoleic acids^37,38^.

In women, BMI variability was not an independent risk factor for T2DM. Therefore, in women, BMI slope predicts T2DM better than BMI variability. In line with our study, in a six-year follow-up of US women aged 18–40, weight variability was not significantly associated with incident T2DM when adjusted for BMI change or the ultimate BMI ^39^. In contrast, in a study on women aged 55–69 years, significant weight variations increased the risk of T2DM incidence^40^; however, the assessment was based on self-recalled weight and self-report incidence of T2DM. The inconsistent results may be attributed to the disparities in methodology (e.g. inclusion and exclusion criteria, adjustments for confounding factors follow-up duration), lack of a standardized definition for variability, and, primarily, differences in the demographic characteristics of the target populations.

The development of T2DM is inferred by many biological, physiological, environmental, and genetic variables, all indulged with sex differentiation. Therefore, sex disparity observed in the association of BMI variability and development of T2DM might be due to differential sex-related effects of risk factors on cardiovascular and metabolic outcomes e.g. body fat distribution, fat tissues and glucose metabolism, and different single nucleotide polymorphisms (SNPs) responsible for body fat distribution^41,42^.

Variability measurement methods used in most studies are sensitive to the mean values and the number and intervals of measurements. Moreover, in contrast to the simple measurement indices of variability in BMI trends (e.g. CV), BMI slope and RMSE are not correlated. Since BMI has a non-linear trend over time, RMSE is the best index for evaluating long-term BMI changes over time^43,44^. The population-based design and long-term follow-up of a well-defined representative cohort are other noteworthy strengths of our study. Additionally, anthropometrics in our cohort were directly measured by the trained staff compared to recalled and self-reported measures used by previous reports. Also, besides using RMSE, we applied other variability indices e.g. VIM, SD, and CV, and the results were identical. However, we reduced the effects of the possible confounders by controlling for the conventional risk factors of DM; we did not apply possible modifications of confounders throughout the follow-up. Finally, the lack of information on some environmental factors responsible for the observed weight changes, such as lifestyle behaviors and dietary patterns, may have limited the interpretation of our findings.

Our study demonstrates that BMI slope is the significant independent predictor of T2DM in both sexes. The effect of BMI variability on the development of T2DM is modified by sex and baseline BMI. Higher weight variability is associated with a lower risk of T2DM in overweight and obese men independent of baseline BMI and BMI slope. BMI variability in women and baseline BMI in both gender are not associated with the risk of T2DM. The findings suggest that weight loss effectively protects against T2DM and its advantages outweigh the possible disadvantages caused by weight variability over time.

## Supplementary Information

Supplementary Tables.

## Data Availability

The datasets generated and/or analyzed during the current study are not publicly available due institution’s policy but are available from the corresponding author on reasonable request.
