# Supplementary material for: BMI variability and incident diabetes mellitus, Tehran Lipid and Glucose Study (TLGS)
Source: Sci Rep. 2022 Nov 1;12:18370. doi: 10.1038/s41598-022-22817-6 (PMC9626493; doi:10.1038/s41598-022-22817-6)
Supplement: Supplementary file 1 — Supplementary Tables. [file 41598_2022_22817_MOESM1_ESM.pdf]

BMI Variability and Incident Diabetes Mellitus, Tehran Lipid and Glucose Study

Ladan Mehran, Pouria Mousapour, Davood Khalili, Leila Cheraghi, Mohammadjavad Honarvar, Atieh Amouzegar, Fereidoun Azizi

Supplementary Table S1. The mean, SD, and percentile values of BMI variability measures (SD, CV, and VIM)

					Percentiles				
	**Mean**	**SD**	**Minimum**	**Maximum**	**5th**	**25th**	**50th (median)**	**75th**	**95th**
**Male**									
BMI-SD	1.46	1.04	0.03	17.82	0.43	0.82	1.26	1.83	3.12
BMI-CV	5.41	3.38	0.13	39	1.64	3.08	4.68	6.78	11.76
BMI-VIM	6.7	4.46	0.15	62.61	2.02	3.80	5.76	8.33	14.54
**Female**									
BMI-SD	1.76	1.25	0.18	42.57	0.58	1.07	1.55	2.20	3.61
BMI-CV	6.14	3.29	0.62	29.56	2.12	3.82	5.46	7.7	12.52
BMI-VIM	7.60	4.27	0.77	77.65	2.61	4.71	6.74	9.52	15.36

SD, standard deviation; CV, coefficient of variation; VIM, variability independent of the mean

Supplementary Table S2. Sex- and BMI-stratified hazard ratios of type 2 diabetes mellitus according to each unit increase in BMI variability measures

Males

	SD		CV		VIM	
	**HR (%95 CI)**	***P* value**	**HR (%95 CI)**	***P* value**	**HR (%95 CI)**	***P* value**
**BMI<25**						
Unadjusted	1.13 (0.87-1.45)	0.356	1.01 (0.95-1.08)	0.686	1.01 (0.96-1.07)	0.659
Age-adjusted	1.31 (1.06-1.62)	0.012	1.06 (0.99-1.12)	0.063	1.05 (0.99-1.10)	0.056
Model 1	1.27 (0.91-1.78)	0.164	1.06 (0.98-1.16)	0.141	1.05 (0.98-1.13)	0.141
Model 2	1.36 (0.94-1.95)	0.102	1.08 (0.99-1.18)	0.093	1.07 (0.99-1.15)	0.092
**BMI≥25**						
Unadjusted	0.92 (0.76-1.10)	0.349	0.95 (0.89-1.01)	0.105	0.96 (0.91-1.01)	0.115
Age-adjusted	1.02 (0.85-1.21)	0.85	0.98 (0.92-1.04)	0.536	0.99 (0.94-1.03)	0.570
Model 1	0.67 (0.50-0.90)	0.008	0.90 (0.82-0.98)	0.016	0.92 (0.85-0.98)	0.015
Model 2	0.67 (0.50-0.90)	0.008	0.90 (0.82-0.98)	0.016	0.92 (0.85-0.98)	0.015

Females

	SD		CV		VIM	
	**HR (%95 CI)**	***P* value**	**HR (%95 CI)**	***P* value**	**HR (%95 CI)**	***P* value**
**BMI<25**						
Unadjusted	1.03 (0.79-1.34)	0.833	1.00 (0.93-1.08)	0.958	1.00 (0.94-1.06)	0.949
Age-adjusted	1.14(0.88-1.48)	0.308	1.04 (0.97-1.12)	0.303	1.03 (0.97-1.09)	0.302
Model 1	0.50 (0.23-1.09)	0.081	0.90 (0.76-1.07)	0.241	0.92 (0.8-1.05)	0.227
Model 2	0.51 (0.23-1.10)	0.086	0.90 (0.76-1.07)	0.248	0.92 (0.80-1.06)	0.233
**BMI≥25**						
Unadjusted	1.07 (1.03-1.11)	<0.001	1.01 (0.97-1.05)	0.583	1.02 (0.99-1.05)	0.94
Age-adjusted	1.07 (1.04-1.11)	<0.001	1.04 (1.00-1.08)	0.05	1.03 (1.01-1.05)	0.002
Model 1	0.92 (0.78-1.08)	0.311	0.98 (0.93-1.03)	0.414	0.98 (0.94-1.02)	0.405
Model 2	0.93 (0.79-1.09)	0.386	0.98 (0.93-1.04)	0.499	0.98 (0.94-1.03)	0.489

Model 1 is adjusted for age, baseline BMI, and BMI slope.

Model 2 is adjusted for age, baseline BMI and BMI slope as well as family history of diabetes, education level and smoking.

SD, standard deviation; CV, coefficient of variation; VIM, variability independent of the mean; HR, hazard ratio; CI, confidence interval.
